# Impact of the host on *Toxoplasm*a stage differentiation

**DOI:** 10.15698/mic2017.07.579

**Published:** 2017-06-22

**Authors:** Carsten G.K. Lüder, Taibur Rahman

**Affiliations:** 1 Institute for Medical Microbiology, University Medical Center Goettingen, Goettingen, Germany.

**Keywords:** Toxoplasma gondii, stage conversion, bradyzoite, parasite host-interaction, host cell, metabolism, cell cycle, immune response

## Abstract

The unicellular parasite *Toxoplasma gondii* infects warm-blooded animals and humans, and it is highly prevalent throughout the world. Infection of immunocompetent hosts is usually asymptomatic or benign but leads to long-term parasite persistence mainly within neural and muscular tissues. The transition from acute primary infection towards chronic toxoplasmosis is accompanied by a developmental switch from fast replicating and metabolically highly active tachyzoites to slow replicating and largely dormant bradyzoites within tissue cysts. Such developmental differentiation is critical for *T. gondii* in order to complete its life cycle and for pathogenesis. Herein, we summarize accumulating evidence indicating a major impact of the host cell physiology on stage conversion between the tachyzoite and the bradyzoite stage of the parasite. Withdrawal from cell cycle progression, proinflammatory responses, reduced availability of nutrients and extracellular adenosine can indeed induce tachyzoite-to-bradyzoite differentiation and tissue cyst formation. In contrast, high glycolytic activity as indicated by increased lactate secretion can inhibit bradyzoite formation. These examples argue for the intriguing possibility that after dissemination within its host, *T. gondii* can sense its cellular microenvironment to initiate the developmental program towards the bradyzoite stage in distinct cells. This may also explain the predominant localization of *T. gondii* in neural and muscular tissues during chronic toxoplasmosis.

## INTRODUCTION

Developmental switching between life cycle stages is critical for the biology of a large number of unicellular microorganisms. It is also important for the course of infectious diseases caused by bacteria, fungi and protozoa. Examples include differentiation between planktonic and sessile bacteria during biofilm formation 1, between yeast and hyphae and vice versa during infection with distinct *Candida spp.* 2, or between *Anopheles*-transmitted sporozoites and liver stage merozoites of *Plasmodium spp.* during malaria 3. Characterization of those factors that regulate these developmental processes is of fundamental interest to understand the biology of microbial cells and the evolution of multicellular organisms. It can also lead to the identification of novel targets for efficient intervening with onset or progression of infectious diseases.

*Toxoplasma gondii* is an obligate intracellular parasite of the phylum *Apicomplexa* which comprises a variety of pathogens of utmost importance for human and animal health. *T. gondii* is widespread throughout the world and threatens the health particularly of immunocompromised individuals or that of fetuses from recently infected pregnant women 4. In otherwise healthy adults, primary infection with *T. gondii* is regularly controlled by the ensuing Th1-type cell-mediated immune response but nevertheless gives rise to chronic persistence of the parasite, possibly for the host’s life. The parasite’s ability to persist for extended periods of time is critical for transmission between hosts. Its appropriate regulation likely was and still is subject of selective pressure during evolution.

The life cycle of *T. gondii* is complex with members of the *Felidae* and warm-blooded vertebrates, i.e., mammals and birds, as final or intermediate hosts, respectively, and different phases of sexual and asexual reproduction. Not surprisingly, it also involves different parasite stages with sexual gametocytes, transmissible sporozoites, rapidly proliferating tachyzoites and dormant bradyzoites being the most prominent ones. Transition between the different stages requires extensive morphological remodeling, expression of distinct transcriptomes and proteomes, as well as several physiological adaptations 5,6. The differentiation of tachyzoites to bradyzoites within intermediate

hosts is experimentally amenable after *in vitro* infection of different host cells and after *in vivo* infection of laboratory or farm animals 5,7,8,9,10. It is also of major biological and medical interest because the transition between tachyzoites and bradyzoites correlates with the acute or chronic phase of toxoplasmosis, respectively.

**Figure 1:**
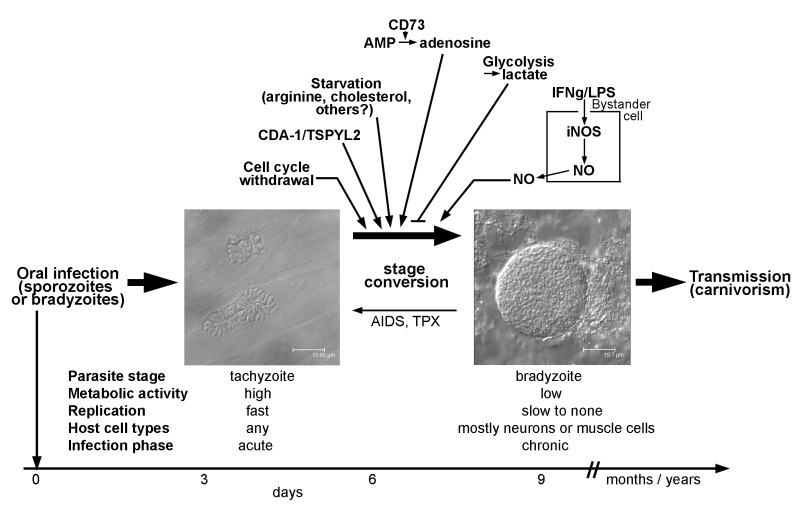
FIGURE 1: *T. gondii* stage conversion and its regulation by the host (cell) microenvironment. Within one to two weeks of infection, highly active tachyzoites (two parasitophorous vacuoles within human foreskin fibroblasts are depicted; left micrograph) convert to relatively dormant bradyzoites (a tissue cyst from mouse brain containing hundreds of individual bradyzoites is shown; right micrograph). Formation of long-lived bradyzoite-containing tissue cysts is crucial for parasite transmission to new hosts. Reconversion of bradyzoites to tachyzoites can occur in immunocompromised individuals (e.g. those with AIDS or transplant recipients (TPX)) leading to life-threatening disease. Distinct characteristics of the infected host cell or of bystander cells can trigger or inhibit differentiation towards the bradyzoite stage as indicated in the upper part of the figure. Noteworthy, parasite-intrinsic triggers may also govern stage differentiation (not depicted). See main text for further details.

Tachyzoites of *T. gondii* develop after oral transmission of infective sporozoites or bradyzoites to intermediate hosts, presumably within the intestinal epithelium (Fig. 1). They rapidly divide within a membrane-bound intracellular compartment, i.e. the parasitophorous vacuole (PV) by a process called endodyogeny 11. The doubling time of this stage depends on the parasite strain and can be as fast as ~5 hours in type I strains but takes 6 - 12 hours in type II and III strains 12. Tachyzoites are metabolically highly active indicating a high demand for nutrients from the environment. After 5 to 6 cell divisions, tachyzoites egress from the host cell in a Ca^2+^-dependent manner and can then infect neighboring host cells 13,14. *T. gondii* invades its host cell by a parasite-driven process that depends on the parasites’ actin-myosin machinery and the discharge of microneme and rhoptry proteins from the apical complex 15. Importantly, the active host cell infection enables *T. gondii* to invade any nucleated cell of its host. Infection of monocytes and dendritic cells particularly contributes to the dissemination of the parasite throughout the hosts’ body 16,17. A sustained parasite replication with tissue damage due to host cell lysis and/or the ensuing cell-mediated immune response correlates with the acute phase of infection and possibly overt disease under certain circumstances. In most cases, however, vigorous cell-mediated immunity is able to restrict tachyzoite replication and to even kill tachyzoites in a timely fashion thereby preventing overt symptoms.

Bradyzoites of *T. gondii* differentiate from tachyzoites within 6 - 9 days after oral infection of mice with oocysts or bradyzoites (Fig. 1) 11, but they can develop as early as 3 days after infection with tachyzoites 18. Bradyzoites are also located inside a PV which, however, over time matures to a so-called intracellular tissue cyst. The most prominent feature of tissue cysts is the development of a thick cyst wall that is thought to confer structural rigidity 19. Tissue cysts may contain several hundreds of bradyzoites which are generally slowly to non-replicating and metabolically rather inactive 5,11, but which can show episodic growth and replication 20. Tissue cysts are long-lived although recent evidence suggests that they rupture at low but continuous frequency 21,22. They are preferentially located in brain and muscular tissue, but they can be found in other organs as for instance liver and kidneys as well 11. Tissue cysts persist for extended periods of time and are hence ideal for transmission of *T. gondii* to new hosts via carnivorism. Re-differentiation of bradyzoites to fast-replicating tachyzoites in immunocompromized patients, i.e. those with AIDS, leads to life-threatening reactivated toxoplasmosis, mostly presenting as *Toxoplasma* encephalitis 4.

Stage conversion between tachyzoites and bradyzoites is an area of intensive research. Both stages have been extensively characterized at the morphological and molecular level (reviewed in 6,11). Additionally, important mechanisms of its developmental biology have been discovered within the last decade 23. What remain less clear is how stage conversion is initiated and which role the host plays in this process. Answering this question may provide clues also for another intriguing issue; that is why tissue cysts are predominantly located in neuronal and muscular tissues. In this review we are summarizing recent evidences that the host cell physiology may determine whether *T. gondii *starts to differentiate from the tachyzoite to the bradyzoite stage or not*.*

## HOST CELL FACTORS REGULATING STAGE CONVERSION 

### Cell type

Despite the ability of *T. gondii* to infect any nucleated cell of intermediate hosts, tissue cysts during chronic toxoplasmosis are not randomly distributed. The preferred localization appears to differ between host species 11. They nonetheless show a predilection for neural and muscle tissues, including brain, eye, skeletal muscle and heart. It must be stressed, however, that *T. gondii* tissue cysts can develop in many different organs including liver and kidney. Within the brain, neurons are the main cell type that harbor tissue cysts during chronic toxoplasmosis 24,25,26. The preferred cell type of tissue cyst development within other organs *in vivo* has not yet been determined.

Two main hypotheses have been proposed to explain the predominant localization in neural and muscular tissues; these are not mutually exclusive. According to the ‘immune privilege-based survival’ hypothesis bradyzoite-containing tissue cysts randomly form in (almost) all parasitized host cell types, but are subsequently eradicated in most tissues except immune-privileged organs including brain, eye and muscle tissues 27. Whereas muscle tissue is not considered a classical immune-privileged organ, skeletal muscle cells (SkMCs) nonetheless display several characteristics of reduced immune functions under physiological conditions 28. The ‘cell type-specific triggering’ hypothesis proposes that bradyzoite differentiation and tissue cyst formation are predominantly triggered in neural and muscular cells thus explaining the predilection for these tissues 29.

Neurons and mature skeletal muscle cells, i.e. myotubes, are post-mitotic, highly differentiated and long-living cells that cannot reenter productive cell cycle 30,31,32. They therefore appear well suited to serve as host cells for a latent parasite stage that needs to survive for extended periods of time. Reasons for a predominant interaction of *T. gondii* with neurons in the brain came recently from elegant analyses of Cre reporter mice infected with Cre-secreting and fluorescently labelled parasites 26. The results suggest that the higher frequency of neurons in the brain as compared to astrocytes and their larger cell surface favor infection of neurons rather than astrocytes. In addition, activation of astrocytes by IFN-γ further reduces the number of infected astrocytes either by killing of intracellular parasites or by preventing initial *Toxoplasma*-astrocyte interactions 26. An impact of IFN-γ-dependent signaling in astrocytes on the distribution of tissue cysts was recently confirmed using mice with a specific depletion of STAT1 in astrocytes 33. In contrast to astrocytes which are able to kill intracellular parasites in an IFN-γ-dependent fashion 34,35, neurons do not restrict *T. gondii* replication after activation with IFN-γ and/or TNF *in vitro* 36. Thus, the predominant localization of *T. gondii* tissue cysts in neurons may result from the likelihood to infect this cell type and the absence of anti-parasitic effector mechanisms.

**Figure 2:**
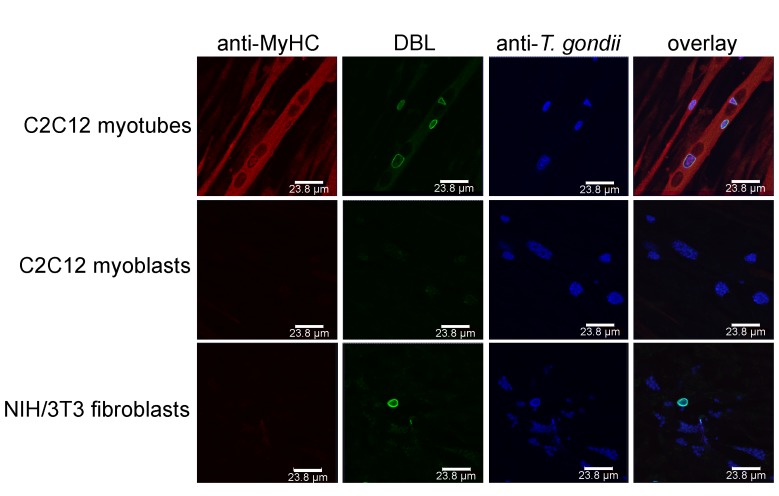
FIGURE 2: *T. gondii *tissue cyst formation is supported by mature syncytial myotubes but not by proliferating myoblasts or fibroblasts. C2C12 murine skeletal muscle cells (SkMCs) were differentiated in vitro to myotubes. Myotubes, myoblasts and fibroblasts were infected with *T. gondii* for 72 hours. Efficient myotube formation was verified by staining with an antibody against myosin heavy chain (MyHC), a marker of differentiation to mature SkMCs (red fluorescence). Total parasites irrespective of the parasite stage were labelled with an antiserum (blue fluorescence) and *T. gondii* tissue cysts were labelled with *Dilichos biflorus* lectin (DBL) recognizing the tissue cyst wall (green fluorescence). Representative images were recorded by confocal laser scanning microscopy.

In addition, distinct cell types are more suitable to sustain tissue cyst formation after infection than others. Neurons and SkMCs readily sustain bradyzoite differentiation *in vitro* without the need of applying exogenous stress 29,37,38. Intriguingly, not only the cell type but rather the state of the host cell appears to be critical. Using mouse SkMCs we recently showed that *T. gondii* readily differentiates to the bradyzoite stage in mature, myosin heavy chain (MyHC)-positive, syncytial myotubes, but not in proliferating, MyHC-negative precursors, i.e. in myoblasts or in fibroblasts (Fig. 2) 39. This strongly suggests that the cellular microenvironment within differentiated myotubes may trigger a developmental pathway of *T. gondii* that leads to conversion to the persistent parasite stage. We thus propose that the ability of SkMCs to terminally differentiate rather than cell type characteristic(s) per se regulates the preferred localization of *T. gondii* tissue cysts in skeletal muscles. It is appealing to assume that terminal differentiation of neurons also triggers bradyzoite development, but this needs future experimental proof. Since myoblasts and myotubes differ in the expression of ~6.500 genes (Rahman & Lüder, unpublished), identification of host molecules/pathways triggering stage conversion in *T. gondii* is a challenge, but relevant factors are beginning to emerge (see below).

### Cell cycle

First evidence that the host cell cycle may regulate stage conversion in *T. gondii* came from observations that forced expression of the human cell division autoantigen (CDA)-1 promotes bradyzoite formation in type II and III strains (Fig. 1) 40. CDA-1 (also known as testis-specific Y-encoded-like 2 (TSPYL2) or differentially expressed nucleolar transforming growth factor-beta 1 target (DENTT)) inhibits cell growth and proliferation by inducing the cell cycle inhibitor p21^Waf1/Cip1^ in a p53- and ERK1/2-dependent manner 41,42. Pharmacological induction of CDA-1 in human-derived fibroblasts using a trisubstituted pyrrole (designated compound 1) or its ectopic expression in epithelial cells indeed inhibits parasite division and induces *T. gondii* bradyzoite antigen (BAG)-1 expression and formation of a cyst wall 40. Consequently, knock-down of CDA-1 prevents the compound 1-induced reduction in parasite division and abolishes BAG-1 expression. Reduced parasite replication 43, expression of stage-specifically expressed BAG-1 (also designated BAG-5) 44,45 and formation of a cyst wall 11 are characteristics of the bradyzoite stage of *T. gondii*. Thus, the above findings indicate that host CDA-1 does suffice to trigger stage conversion in *T. gondii*. Importantly, CDA-1 has recently been proved to play a role in bradyzoite differentiation also in a more physiological environment of *T. gondii* stage conversion 39. First, these authors provided direct evidence that the host cell cycle impacts *T. gondii* development. Thus, differentiation of the murine myoblast cell line C2C12 to polynucleated mature myotubes (see above) is accompanied by up-regulation of mRNA for CDA-1/TSPYL2, and by a halt in host cell cycle progression. As observed in mature SkMCs *in vitro* and *in vivo*, such differentiation and withdrawal from the host cell cycle induce *T. gondii* to convert from the tachyzoite to the bradyzoite stage (Fig. 1; see also above). Remarkably, shRNA knock-down of CDA-1/TSPYL2 in C2C12 SkMCs abolishes the increased bradyzoite formation as observed in mature myotubes and allows vigorous parasite replication 39. It must be stressed that knock-down of CDA-1/TSPYL2 also prevents formation of myotubes suggesting multiple effects on the host cell including sustained cell cycle progression, inhibition of muscle cell differentiation and others. It nevertheless clearly links the withdrawal from the host cell cycle to stage differentiation in *T. gondii*. CDA-1/TSPYL2 expression and permanent arrest of mature SkMCs in a G_0_ phase of the cell cycle could thus provide an appropriate signal in muscle tissue *in vivo* to trigger *T. gondii* stage conversion. Intriguingly, in mice, CDA-1/TSPYL2 is highly expressed in different regions of the brain, including cerebral cortex and hippocampus and it is expressed to a lower extent also in the gonads 46,47. It will thus be interesting to see whether CDA-1/TSPYL2 and cell cycle inhibition also triggers *T. gondii* stage conversion in the brain. The mechanism of how CDA-1 regulates bradyzoite differentiation is currently unknown.

### Pro-inflammatory immune responses

Pro-inflammatory molecules were among the first host-derived signals for which pro-bradyzoite activities have been confirmed. Activation of murine macrophages with IFN-γ and/or LPS efficiently induces differentiation of *T. gondii* to the bradyzoite stage (Fig. 1) 48,49. The level of macrophage activation is critical, since only intermediate levels of activation induce expression of bradyzoite-specific antigens 49. This was explained by findings that only parasites that show limited replication but not a complete halt of replication are able to differentiate towards the bradyzoite stage 49. Later on it was confirmed that transition through a G2-related cell cycle phase prior to mitosis and cytokinesis is required for *T. gondii* to enter the differentiation program to the bradyzoite stage 43. Thus, non-activated host macrophages favor direct S phase-to-mitosis progression indicative for the tachyzoite cell cycle 12, while too strongly activated macrophages prevent *T. gondii* reaching a putative G2-associated checkpoint that may be required for initiating the developmental program towards bradyzoite formation. Bradyzoite formation in activated macrophages correlates with nitric oxide (NO) production by the inducible NO synthase (iNOS, also designated NOS2), and the NO donor sodium nitroprusside (SNP) partially mimics activation by IFN-γ/LPS 49. The underlying mechanism of NO-mediated stage conversion in *T. gondii* seems to partially involve NO reacting with iron-sulfur centers of proteins of the host cells mitochondrial electron transport chain 49. It is interesting to note that this links macrophage activation and its impact on bradyzoite formation in *T. gondii* with host cell metabolism (see also below). Since SNP even at high concentrations, however, only partially mimics the effect of macrophage activation, mechanisms others than the direct toxicity of NO appear to also impact bradyzoite formation. Induction of reactive oxygen species and starvation of *T. gondii* for tryptophan by activation of the oxidative burst 50 or the indoleamine 2,3-dioxygenase (IDO) 51,52, respectively, are possible candidates, but this needs to be validated. Furthermore, since iNOS catalyzes the production of NO by converting arginine to citrulline, and since *T. gondii* is auxotrophic for arginine 53,54, arginine limitation can also contribute to inducing *T. gondii* stage conversion in activated macrophages.

Other cell types including human foreskin fibroblasts (HFF), murine astrocytes or rat neurons, astrocytes and microglia do not increasingly support the differentiation from tachyzoites to bradyzoites after activation with IFN-γ 37,40,55,56,57. This might be due to inappropriate activation of iNOS in these cells since SNP-derived exogenous NO does induce bradyzoite formation in human fibroblasts or rat brain cells to various extents 37,58,59. In human fibroblasts, IL-6 also induces differentiation towards bradyzoites and formation of cyst-like structures 55. Together, these data indicate that pro-inflammatory signals from the host cell can trigger stage conversion in *T. gondii* in a cell type- and context-dependent fashion. The significance of these findings for the course of infection *in vivo* is less clear. Detection of bradyzoites *in vivo* coincides with development of a vigorous cell-mediated immune response and this has supported the view that pro-inflammatory signals may trigger stage conversion in the infected host. Additionally, immunological competence is clearly required to prevent reactivation of latent tissue cysts during chronic toxoplasmosis in humans 4,60 and mice 61,62,63. However, those cell types supporting bradyzoite formation after activation with pro-inflammatory cytokines are not preferred host cells for parasite persistence *in vivo*, and those which harbor latent tissue cysts during chronic infection do obviously not support bradyzoite formation in response to pro-inflammatory signals *in vitro*. Direct evidence for activation of host cells by pro-inflammatory molecules triggering stage conversion *in vivo* is thus lacking.

A possible scenario is, however, a bystander effect of activated inflammatory macrophages or other immune cells triggering stage conversion in adjacent *Toxoplasma*-infected non-hematopoietic cells including neurons and muscle cells via diffusion of NO (Fig. 1). Alternatively, activated macrophages could also trigger formation of bradyzoite-containing tissue cysts which can then infect neighboring cells via migration of free bradyzoites or even of tissue cysts 5. Both scenarios await nevertheless future verification. It is nevertheless generally accepted that cell-mediated immunity is necessary to stabilize tissue cysts and to prevent reactivation 4,60,61. This is supported by the finding that murine astrocytes support long-term cultivation of tissue cysts *in vitro* 57.

### Host stress response

Applying extracellular stress including alkaline pH, heat shock or treatment of infected host cells with toxic substances as for instance sodium arsenite or mitochondrial inhibitors can induce bradyzoite differentiation and/or tissue cyst formation *in vitro* (reviewed in 29). Whereas these stressors are artificial they have nonetheless helped to study *T. gondii* stage differentiation *in vitro*. In the context of this review their ability to induce stage differentiation raises the question whether they directly act on the parasite or indirectly via the host stress response or both. Exposure of extracellular parasites to alkaline pH or to SNP prior to infection of host cells in the absence of exogenous stress triggers bradyzoite development 58. However, the extent to which bradyzoite formation is induced under these conditions is lower as compared to treatment of infected host cells with these stressors 58. Whereas it is difficult to draw robust conclusions from these data due to different length of treatments, they nevertheless might suggest that stage conversion in *T. gondii* is both a direct parasite response to stress and an indirect effect mediated by the host cell. Whether or not a host stress response can trigger stage differentiation *in vivo* awaits clarification.

### Metabolic features

The fast replicating tachyzoites rely on higher nutrient uptake from the host cell than the more quiescent bradyzoites in order to satisfy their demands for energy and metabolic building blocks. *T. gondii* is auxotrophic for several metabolites including purines, arginine, cysteine 64, those amino acids that are also essential for humans except lysine 64, polyamines, cholesterol, choline and vitamins 53,54. Limited supply of essential molecules and basic nutrients from the host cell may thus restrict tachyzoite replication and thereby favor developmental switching towards the bradyzoite stage (Fig. 1) 43,49. This view has been convincingly confirmed when intracellular parasites were grown in culture medium containing not more than 5-10 µM of arginine 65. It is remarkable that arginine starvation triggers stage conversion to high extent even in a parasite strain that is generally rather refractory to tissue cyst formation (RH strain). However, arginine plasma concentrations in mammals including humans vary between 100 and 250 µM 66 and thus exceed by far those 5-10 µM required to trigger stage conversion in *T. gondii*. In addition, the reported intracellular arginine concentrations of 100 to 1000 µM in human cells 66 are also comparatively high. The intracellular threshold necessary to trigger stage differentiation in *T. gondii* is unknown, but these figures make it rather unlikely that arginine can drop *in vivo* to levels that suffice to trigger bradyzoite formation. Starvation of *T. gondii* for multiple nutrients might however suffice to favor stage differentiation. For instance, depletion of lipoproteins from culture medium also induces stage conversion which is abolished by addition of low-density lipoproteins (LDL) 67. Since LDL particles are major cholesterol carriers in the blood, and since *T. gondii* is auxotrophic for LDL-derived cholesterol 68, the authors concluded that starvation of LDL-derived cholesterol can indeed trigger bradyzoite formation 67. Whether limited availability of LDL-cholesterol regulates stage conversion *in vivo* needs experimental validation. It is noteworthy that infection of Chinese hamster ovary cells with *T. gondii* tachyzoites increases LDL uptake by threefold 68 suggesting a particularly high demand for cholesterol of this parasite stage that cannot be satisfied by the regular cholesterol uptake of these cells.

Ambient CO_2_ concentration (0.03%) in the environment triggers bradyzoite formation and tissue cyst formation in a parasite strain that is prone to stage conversion *in vitro* and *in vivo* (Prugniaud strain) 5. *T. gondii* predominantly synthesizes pyrimidines de novo from amino acids, bicarbonate and 5-phosphoribosyl-1-pyrophosphate 53. Low CO_2_/bicarbonate may therefore limit the availability of pyrimidines and hence inhibit DNA and RNA synthesis by *T. gondii*. Due to CO_2_ concentrations in human blood between 4.5 to 5.8% at standard atmospheric pressure it is however unlikely that CO_2_/bicarbonate availability is a trigger of stage conversion under physiological conditions.

Contrary to the examples above where starvation of *T. gondii* triggers bradyzoite formation, distinct metabolites the parasite needs for optimal growth can also trigger bradyzoite formation. The 5’-ecto-nucleosidase (CD73) is expressed on the surface of various mammalian cells including CNS cells and dephosphorylates adenosine monophosphate (AMP) to adenosine. This in turn dampens excessive inflammation via adenosine receptor signaling or can be used for purine salvage. *T. gondii* is unable to synthesize purines *de novo* (see above) 53,54 and adenosine is its major source for purines 69. Intriguingly, CD73-deficient mice show reduced bradyzoite differentiation and reduced tissue cyst burden in their brains (Fig. 1) and consequently a decreased mortality as compared to wild type mice 70. Reduced bradyzoite and tissue cyst formation in stressed CD73^-/-^ astrocytes can be rescued with exogenous adenosine. Furthermore, adenosine receptor signaling is dispensable for triggering stage differentiation by adenosine. Thus, external adenosine can trigger stage differentiation in *T. gondii* probably after its uptake from the environment. Whether bradyzoites have an unexpectedly high demand for adenosine or whether it functions as a signaling molecule to trigger stage conversion awaits future clarification.

A link between lactate production by different host cells and differentiation of *T. gondii* has been established 71. Indeed, increased glycolytic activity as evidenced by secretion of lactate correlates with the inability of NIH/3T3 fibroblasts and 293T embryonic kidney cells to sustain bradyzoite formation under stress conditions. Supernatants from these cells inhibit bradyzoite formation also in HFF fibroblasts and Vero kidney cells which are normally permissive for bradyzoite formation. Furthermore, forced activation of glycolysis in HFF and Vero cells by expression of a myristoylated, i.e. an active version of the Akt kinase and/or by increasing glucose levels also inhibit stage conversion 71. The data thus suggest that high glycolytic activity of the host cell provides nutrients to the parasites that suffice to sustain the high metabolic demands of tachyzoites thereby inhibiting bradyzoite formation (Fig. 1). They also provide compelling evidence that metabolic features of the host cell can indeed impact stage conversion in *T. gondii*. It must be stressed that lactate itself accounts for only 30% of the inhibitory activity of 293T supernatant and that it only inhibits bradyzoite formation in Vero cells but not in HFF cells 71. Thus, HFF and Vero cells obviously differ in their regulation of glycolytic activity by external lactate. It is also important to note that increased glycolysis only prevents stress-induced stage conversion. Whether it can also inhibit spontaneous stage conversion as observed in distinct cell types (see above), or whether low glycolytic activity of the host cell does suffice to facilitate stage conversion in distinct cells needs to be clarified.

Collectively, these data provide clear evidence that distinct metabolic characteristics of the host cell can regulate stage conversion in *T. gondii*.

## CONCLUSIONS

The data discussed above provide strong evidence that the host cell microenvironment regulates stage differentiation in *T. gondii*. Host cell cycle, host cell metabolism and inflammatory responses, the latter possibly by acting on infected non-immune bystander cells, clearly affect bradyzoite formation *in vitro*. Distinct features as for instance the CD73-mediated adenosine formation have been proved to regulate bradyzoite and tissue cyst formation also *in vivo* (see above). We thus propose that differentiation towards the bradyzoite stage can occur spontaneously when the parasite encounters an appropriate host cell environment. Terminally differentiated SkMCs and neurons may represent such a suitable cellular niche and this may explain the predominant localization of tissue cysts in muscular and neural tissues during chronic toxoplasmosis. Inflammatory responses including NO production may additionally limit parasite replication and hence promote stage differentiation. Following infection with sporozoites or bradyzoites, *T. gondii* can also spontaneously slow down its cell cycle and differentiate to the bradyzoite stage after ~20 divisions of rapid tachyzoite growth, i.e. via a programmed developmental pathway 43,72. The factors regulating tachyzoite-to-bradyzoite differentiation are thus likely diverse and complex.

From the findings discussed in this review several important questions emerge. Firstly, do host cell cycle, host cell metabolism and inflammatory responses act independently on *T. gondii* to initiate bradyzoite differentiation? Secondly, what are the host cell signals that are perceived by the parasite and how are these transduced into the program that ultimately leads to stage conversion? And finally, do the same signals trigger stage conversion within different host cell types (and/or different host species) and does this indeed explain the predilection of *T. gondii* to persist in certain tissues, i.e. brain and muscle? Elucidating these issues is critical to better understand the developmental processes in *T. gondii* and possibly also in other *Apicomplexa*.
